# Self-Efficacy Measured Through the Italian Diabetes Management Self-Efficacy Scale (IT-DMSES) in Individuals with Type 2 Diabetes

**DOI:** 10.3390/jcm15145626

**Published:** 2026-07-17

**Authors:** Santo Colosimo, Anna Guerrini Usubini, Sara Paola Mambrini, Gabriele Amodeo, Alessandro Sartorio, Gianluca Castelnuovo, Simona Bertoli

**Affiliations:** 1 Department of Food, Environmental and Nutritional Sciences (DeFENS), University of Milan, 20133 Milan, Italy; 2 Laboratory of Nutrition and Obesity Research, Istituto Auxologico Italiano IRCCS, 28824 Piancavallo-Verbania, Italy; 3 Psychology Research Laboratory, Istituto Auxologico Italiano, Istituto di Ricovero e Cura a Carattere Scientifico (IRCCS), 28824 Piancavallo-Verbania, Italy; 4 Istituto Auxologico Italiano IRCCS, 28824 Piancavallo-Verbania, Italy; 5 Experimental Laboratory for Auxo-Endocrinological Research, Istituto Auxologico Italiano, Istituto di Ricovero e Cura a Carattere Scientifico (IRCCS), 28824 Piancavallo-Verbania, Italy; 6 Department of Psychology, Catholic University of Milan, 20123 Milan, Italy

**Keywords:** type 2 diabetes, self-efficacy, Italian Diabetes Management Self-Efficacy Scale, obesity

## Abstract

**Background:** Obesity and Binge Eating Disorder (BED) are common comorbidities in type 2 diabetes mellitus (T2DM), often complicating disease management. Self-efficacy—the belief in one’s ability to manage diabetes—has been shown to influence self-care behaviours and outcomes. This study aimed to assess diabetes management self-efficacy among adults with obesity and T2DM undergoing multidisciplinary rehabilitation, focusing on the impact of BMI, BED, and gender. **Methods:** A cross-sectional study was conducted with 137 adults (BMI ≥ 30 kg/m^2^) with T2DM admitted to a residential rehabilitation center. Self-efficacy was measured using the Italian Diabetes Management Self-Efficacy Scale (IT-DMSES), which evaluates confidence in lifestyle and disease management tasks. Data on BMI, BED status, age, and gender were collected. **Results:** The mean IT-DMSES score was 116.7 ± 23.7, with females scoring slightly higher than males (120.3 ± 24.8 vs. 112.5 ± 22.0, *p* = 0.057). Higher BMI was significantly associated with lower self-efficacy, particularly in lifestyle (R = 0.282, *p* = 0.002) and disease management (R = 0.271, *p* = 0.002) domains. Several individual items, including those related to dietary control and medication adherence, showed strong negative correlations with BMI. BED independently predicted lower self-efficacy on item 13 (emotional eating) (Estimate = −3.18, *p* = 0.001), even after adjusting for BMI, age, and gender. No significant interactions between gender and BED were observed for overall self-efficacy or domain scores. **Conclusions:** Although the exploratory nature of the findings requires confirmation, our results underline the independent association of low self-efficacy with BED.

## 1. Introduction

Type 2 diabetes mellitus (T2DM) and obesity are two intricately linked conditions that together represent a major global health challenge [1], characterized not only by physical complications but also by profound behavioural and psychological determinants of disease outcomes. Effective management of diabetes relies heavily on sustained self-care behaviours [2], with self-efficacy recognized as a core psychological factor influencing individuals’ capacity for successful disease management [1]. Emerging evidence suggests that individuals with severe obesity may encounter pronounced barriers to diabetes self-management [3], leading to reduced confidence in carrying out essential self-care activities such as dietary regulation, physical activity, and glucose monitoring. The recent literature also highlights the additional burden posed by comorbid Binge Eating Disorder (BED), which has been associated with poorer metabolic outcomes, maladaptive eating behaviours, and lower perceived ability to manage diabetes-related tasks [4,5].

While several studies have explored the relationship between self-efficacy and diabetes management in community or outpatient populations, far less attention has been devoted to individuals with severe obesity undergoing inpatient multidisciplinary rehabilitation. This clinical setting represents a particularly relevant yet understudied context, as patients admitted to rehabilitation programmes often present with more advanced disease, higher levels of psychological distress, and complex eating behaviours that may substantially affect diabetes self-management. Moreover, inpatient rehabilitation offers an intensive therapeutic environment in which behavioural change is actively targeted, making the assessment of self-efficacy particularly informative for tailoring individualized interventions [6]. Despite the known negative impact of excess adiposity and disordered eating on clinical outcomes in T2DM, research specifically addressing self-efficacy in managing diabetes patients with concomitant severe obesity and BED remains sparse, especially within Italian inpatient rehabilitation settings.

In response to this knowledge gap, the current study aims to investigate self-efficacy in managing diabetes among adults with T2DM and severe obesity, using the Italian version of the Diabetes Management Self-Efficacy Scale (IT-DMSES). By examining the relationships among BMI, self-efficacy scores, and the presence of BED, this research seeks to elucidate the specific behavioural and psychological challenges faced by this vulnerable subgroup, thereby informing the development of targeted interventions to support effective diabetes management.

## 2. Materials and Methods

### 2.1. Participants and Procedures

This preliminary study employed a cross-sectional design to investigate self-efficacy in managing diabetes among patients with type 2 diabetes mellitus (T2DM). Participants were recruited at Istituto Auxologico Italiano IRCCS, Piancavallo (VB), a third-level clinical center for multidisciplinary obesity rehabilitation between June and October 2025. Inclusion criteria were as follows: (1) being Italian; (2) aged greater than 18 years; (3) having a body mass index, BMI, ≥30 (Kg/m^2^); and (4) diagnosis of T2DM. Exclusion criteria included cognitive impairments or psychiatric conditions that could interfere with questionnaire completion. All participants provided written informed consent prior to enrollment. Once enrolled, participants were asked to provide their socio-demographic data and complete a self-report questionnaire. The questionnaire was administered in a quiet setting to minimize distractions, and research staff were available to clarify any questions.

This study was conducted in accordance with the ethical principles outlined in the Declaration of Helsinki. The research protocol received prior approval from the Ethical Committee Lombardy 5, Milan, Italy (date of approval: 20 May 2025; research code: 43C501), and all procedures complied with applicable regulatory requirements.

### 2.2. Measures

Socio-demographic data (e.g., gender, age) were self-reported by participants. Other clinical characteristics (weight, height, BMI, comorbidities including Binge Eating Disorder, current medications) were collected from medical records.

The diagnosis of BED had been established during the clinical assessment performed at admission, based on a clinical interview conducted according to the Diagnostic and Statistical Manual of Mental Disorders, Fifth Edition (DSM-5) criteria. In addition, the diagnosis was supported by the administration of the Binge Eating Scale (BES; [7]), a validated self-report questionnaire assessing the severity of binge eating symptoms and associated psychopathological features.

Self-efficacy was assessed using the IT-DMSES [8]. This validated self-report questionnaire assesses individuals’ confidence in engaging in diabetes self-management activities (e.g., checking blood sugar). It consists of 15 items scored on a 0–10 point numerical scale, exploring two domains: “Disease management” and “Lifestyle management”. Higher scores indicate higher self-efficacy levels. The Italian version of the questionnaire showed good psychometric properties with high reliability for the Disease Management (α = 0.849) and excellent reliability for the Lifestyle Management (α = 0.900) subscales.

### 2.3. Statistical Analysis

Descriptive statistics were calculated as means and standard deviations for continuous variables and as frequencies with 95% confidence intervals for categorical variables. Group comparisons by gender at baseline were conducted using the *t*-test or the χ^2^-test for independent samples. Univariate linear regression analyses were performed to examine associations between BMI, considered as the independent variable, and each IT-DMSES item and domain as dependent variables. Corrections for confounders (age and gender) was performed through multivariate linear regression analysis, and data are available in the Supplementary Materials. Subjects with incomplete data were excluded from the analyses. A two-sided *p*-value of less than 0.05 was considered statistically significant for all analyses. The data were analyzed using IBM SPSS Statistics, version 28 (IBM Corp., Armonk, NY, USA).

## 3. Results

A total of 137 participants completed the IT-DMSES. The mean age was 64 (10.3) for males (*n* = 73) and 64.9 (11.3) for females (*n* = 64); age was similar in the two genders (*p* = 0.652) (Table 1). The mean BMI was 39.3 (8.3) for males (*n* = 73) and 43.1 (8.6) for females (*n* = 64); BMI values were statistically different in the two genders (*p* = 0.012). The mean total IT-DMSES score for the entire sample was 116.7 (SD = 23.7) (Table 1). Male participants (*n* = 64) had a lower mean total IT-DMSES score (112.5, SD = 22.0) compared to female participants (*N* = 73; mean = 120.3, SD = 24.8), although this difference did not reach statistical significance (*p* = 0.057) (Table 1).

For the individual IT DMSES items, only item 5 (“keep my weight under control”) showed a statistically significant difference between genders (males: 6.3 ± 2.5; females: 5.0 ± 2.8; *p* = 0.008) (Table 1). No other items or composite scores demonstrated statistically significant sex differences. The composite for IT-DMSES Lifestyle Modification was 6.0 ± 2.0 (males: 6.3 ± 2.0; females: 5.7 ± 2.0; *p* = 0.160). For Disease Management, the mean score was 7.8 ± 1.8 (males: 7.9 ± 1.8; females: 7.6 ± 1.9; *p* = 0.345) (Table 1).

Univariate linear regression analyses were conducted to examine the association between BMI and scores on each IT-DMSES item. The results indicated that several IT-DMSES items were significantly and negatively associated with BMI (Table 2).

Specifically, higher self-efficacy scores on items 1 (R = 0.259, Estimate = −0.738, *p* = 0.002), 2 (R = 0.217, Estimate = −0.571, *p* = 0.012), 5 (R = 0.354, Estimate = −1.13, *p* = 0.001), 7 (R = 0.251, Estimate = −0.743, *p* = 0.003), 8 (R = 0.267, Estimate = −0.960, *p* = 0.002), 9 (R = 0.272, Estimate = −0.825, *p* = 0.002), and 13 (R = 0.234, Estimate = −0.668, *p* = 0.007) were all significantly correlated with lower BMI values. Items 3, 4, 6, 10, 11, 12, 14, and 15 did not show statistically significant associations with BMI (all *p* > 0.05).

At the domain level, both the IT-DMSES Lifestyle Modification (R = 0.282, Estimate = −1.25, *p* = 0.002) and Disease Management (R = 0.271, Estimate = −1.31, *p* = 0.002) composite scores demonstrated significant negative associations with BMI. The total IT-DMSES score was associated with BMI, but did not reach significance (R = 0.159, Estimate = −0.0577, *p* = 0.064).

Even after adjustment for age and gender, a multivariate regression analysis showed similar significance for the linear association of DMSES scores and BMI, with the exception of items 13 and 15 (Supplementary Table S1).

Furthermore, in a multivariable regression model that included gender, BED, age, and BMI as predictors, the presence of BED was independently associated with lower scores on IT-DMSES item 13 (Estimate = −3.18, SE = 0.88, *p* = 0.001) (Table 3). No significant interactions between gender and BED were observed for total IT-DMSES, lifestyle, or disease management domains (all *p* > 0.05). These findings highlight a specific negative impact of BED and increasing BMI on certain aspects of diabetes-related self-efficacy, over and above the influence of gender stratification.

## 4. Discussion

This exploratory study investigated self-efficacy in managing diabetes among 137 adults with severe obesity and T2DM enrolled in a multidisciplinary rehabilitation programme. Results were controlled for gender, BMI and BED. Importantly, this study focuses specifically on adult and older adult patients with obesity, a population that reflects the demographic characteristics of patients typically admitted to our Institute, consistent with previous observations [9]. Focusing on this group is crucial, as they may face unique physiological, psychological, and social challenges that can negatively impact self-efficacy and, consequently, the effectiveness of diabetes management. Given the exploratory nature of this cross-sectional study, the findings suggest that, although overall diabetes self-efficacy appeared to be high across genders, individuals with higher BMI tended to report lower self-efficacy, particularly in domains related to lifestyle and disease management. These associations remained after adjustment for key demographic and clinical variables. While no causal inferences can be drawn, these findings may indicate that higher BMI is associated with lower perceived confidence in diabetes self-management in this high-risk population. This pattern warrants further investigation in longitudinal and hypothesis-driven studies to clarify the underlying psychological and behavioural mechanisms. In particular, there was a negative association between BMI and several individual aspects of self-efficacy as measured through IT-DMSES.

Specifically, items n. 1, 2, 5, 7, 8, 9, 13 of IT-DMSES showed significant negative correlations with BMI. This result may suggest that participants with higher BMI report lower confidence in performing certain diabetes self-management tasks. Similarly, composite domain scores for Lifestyle Modification and Disease Management were inversely associated with BMI. Conversely, the association between the total score of IT-DSMES and BMI was not significant. However, the observed trend was toward an inverse association, suggesting that higher BMI may be associated with lower overall self-efficacy.

Even if preliminary, these results are consistent with evidence from a recent study, which reported that adults with type 2 diabetes and obesity exhibited lower self-care behaviours and reduced confidence in their ability to manage diabetes compared with individuals with lower BMI [10].

In our multivariable regression models, adding gender, age, BMI, and presence of BED, we found that BED was independently associated with lower self-efficacy in managing diabetes on IT-DMSES item 13 (“*Adjust my eating plan when I am feeling stressed or anxious*”). This specific negative association suggests that binge-eating behaviour can negatively influence confidence in performing specific diabetes management tasks, beyond the influence of BMI or demographic factors such as gender. However, given the cross-sectional design of the study, these findings should be interpreted as an association rather than evidence of a causal relationship. These findings align with and further elaborate on recent international studies that link BED to poorer metabolic and psychological outcomes in the context of diabetes and corroborate evidence that increasing degrees of obesity are associated with greater self-management challenges [11].

The observed negative association between BMI and self-efficacy in diabetes management indicates that higher BMI levels were associated with lower confidence in performing essential self-management behaviours. This association may reflect the greater complexity of diabetes management among individuals with severe obesity, although longitudinal studies are needed to clarify the temporal relationship between obesity severity and self-efficacy. These findings are significant given their clinical implications, particularly because self-efficacy has been consistently identified as a critical determinant of successful diabetes self-management. High levels of self-efficacy in managing diabetes are strongly associated with improved glycemic control, enhanced psychological well-being, and greater adherence to self-care behaviours, as demonstrated by numerous recent studies [12]. A portion of the items in the IT-DMSES specifically targets self-efficacy related to alimentary or dietary management, including behaviours such as choosing healthy foods, following an eating plan, and adjusting diet in response to physical activity or illness (items 4, 7, 8, 10 and 12). In our study population of patients living with obesity admitted to a weight loss rehabilitation program, perceived self-efficacy on these alimentary management items was notably lower. This observed reduction in confidence may be influenced by selection bias, as patients enrolled in such programs typically present more complex challenges with dietary adherence and weight control, reflecting their greater need for intensive intervention and support within this clinical setting. However, this clinical context also represents a unique opportunity for early identification of low self-efficacy and for the implementation of targeted, multidisciplinary interventions aimed at strengthening self-management skills and supporting sustainable lifestyle changes. Thus, reduced self-efficacy may not only reflect greater treatment complexity but may also serve as a clinically relevant indicator to personalize rehabilitation strategies and optimize treatment outcomes.

This interpretation aligns with the IT-DMSES construct, where the Lifestyle Management factor, encompassing diet and exercise self-efficacy, often shows greater variability and is sensitive to the specific clinical characteristics of the patient cohort [8].

Yet, most of the literature to date has focused on the general T2DM population or those with moderate obesity, with far less attention paid to cohorts with simultaneous severe obesity and BED. The present investigation extends knowledge by highlighting that, within an Italian inpatient rehabilitation context, the intersection of severe obesity and BED represents a particularly vulnerable clinical subgroup with disproportionately reduced confidence in their capacity to manage diabetes. Furthermore, the robustness and discriminative power of the Italian Diabetes Management Self-Efficacy Scale (IT-DMSES) reaffirm its psychometric validity and clinical applicability, as previously reported in Italian samples [8]. Several strengths must be noted, including the use of the validated IT-DMSES, which has strong psychometric credentials, and the enrolment of a representative sample from a tertiary centre, which increases the real-world relevance of the findings. The research methodology allowed for the separate identification of the effects of BMI, BED, gender, and age, offering a clear understanding of how these variables interplay in shaping self-efficacy.

There are also several limitations inherent to the study design. First, the cross-sectional nature of the work limits any ability to infer causation between psychological variables and clinical outcomes. Second, the use of self-report measures may have introduced some bias, and the results may not be generalizable to other settings or populations with less severe obesity or better access to resources. In this regard, it is important to point out that the study population consisted of patients undergoing inpatient rehabilitation, who may differ from the broader population of individuals with type 2 diabetes managed in outpatient settings. Patients admitted to rehabilitation programs often present with greater clinical complexity, functional limitations, or a higher burden of comorbidities, which may influence both psychological factors and diabetes self-management. Consequently, the generalizability of our findings to other T2DM populations should be considered with caution.

Also, the exploratory nature of the analyses represents a limitation to the implications for the BED-specific findings. Another important limitation of this study is the lack of data on diabetes duration and glycemic control (e.g., HbA1c), which are relevant determinants of diabetes self-efficacy. Their absence limits the ability to distinguish whether lower self-efficacy is primarily related to obesity-related psychosocial factors or to greater disease burden. Therefore, the association between BMI and self-efficacy should be interpreted cautiously, as it may be partially influenced by unmeasured differences in diabetes severity. Future studies should include these clinical variables to better evaluate the independent role of obesity in diabetes self-management confidence.

Future research should address these limitations through longitudinal designs capable of exploring causality and change over time and through the replication of the study, considering additional influencing variables (e.g., the duration of diabetes). Furthermore, interventions specifically tailored to enhance self-efficacy in individuals with severe obesity and BED should be developed and tested for their impact on both psychological and metabolic outcomes [13]. The promising role of cognitive-behavioural approaches, as well as digital, personalized rehabilitation support, deserves rigorous exploration as potential adjuncts to standard multidisciplinary care [14]. Broader psychometric studies are also warranted to consolidate the utility of the IT-DMSES in varied Italian and international samples.

In conclusion, this study underscores the value of self-efficacy assessment and targeted interventions for those with severe obesity and T2DM, with special attention to the substantial subgroup affected by BED. A systematic approach to identifying and addressing self-efficacy deficits in this population may meaningfully enhance diabetes outcomes and overall quality of life.

## Figures and Tables

**Table 1:** Baseline characteristics of the study population.

	Male *n* = 73	Female *n* = 64	Overall *N* = 137	*p* Value
Age [yrs]	64.0 (10.3)	64.9 (11.3)	64.4 (10.7)	0.652
BMI [kg/m^2^]	39.3 (8.3)	43.1 (8.6)	41.1 (8.6)	0.012
IT-DMSES 1	8.6 (2.7)	8.0 (3.4)	8.3 (3.0)	0.280
IT-DMSES 2	6.9 (3.1)	6.5 (3.4)	6.7 (3.3)	0.451
IT-DMSES 3	7.1 (3.3)	6.8 (3.3)	7.0 (3.3)	0.703
IT-DMSES 4	7.1 (2.8)	7.0 (2.3)	7.0 (2.6)	0.817
IT-DMSES 5	6.3 (2.5)	5.0 (2.8)	5.7 (2.7)	0.008
IT-DMSES 6	6.7 (3.8)	7.4 (3.3)	7.0 (3.6)	0.251
IT-DMSES 7	6.7 (2.9)	6.8 (2.9)	6.7 (2.9)	0.780
IT-DMSES 8	6.3 (2.4)	6.4 (2.4)	6.4 (2.4)	0.937
IT-DMSES 9	6.5 (2.9)	5.5 (2.7)	6.0 (2.8)	0.061
IT-DMSES 10	6.1 (3.0)	5.5 (2.9)	5.8 (3.0)	0.204
IT-DMSES 11	5.8 (2.6)	5.6 (2.8)	5.7 (2.7)	0.816
IT-DMSES 12	5.6 (2.4)	5.3 (2.9)	5.4 (2.7)	0.523
IT-DMSES 13	6.5 (3.1)	5.6 (2.9)	6.0 (3.0)	0.080
IT-DMSES 14	9.3 (1.6)	8.9 (1.5)	9.1 (1.6)	0.150
IT-DMSES 15	9.2 (1.7)	8.7 (1.6)	9.0 (1.7)	0.090
IT-DMSES Total	112.5 (22.0)	120.3 (24.8)	116.7 (23.7)	0.057
IT-DMSES Lifestyle Modification	6.3 (2.0)	5.7 (2.0)	6.0 (2.0)	0.160
IT-DMSES Disease Management	7.9 (1.8)	7.6 (1.9)	7.8 (1.8)	0.345
BED (%)	5 (7.9)	6 (11.8)	11 (9.6)	0.491

Note: BMI: Body Mass Index; IT-DMSES: Italian Version of the Diabetes Management Self-Efficacy Scale. BED: Binge Eating Disorder.

**Table 2:** Unadjusted univariate linear regression model for BMI vs. IT-DMSES items.

Item	R	R^2^	β	*p* Value
IT-DMSES 1	0.259	0.067	−0.74	0.002
IT-DMSES 2	0.217	0.047	−0.57	0.012
IT-DMSES 3	0.136	0.018	−0.36	0.123
IT-DMSES 4	0.133	0.017	−0.45	0.128
IT-DMSES 5	0.354	0.126	−1.13	0.001
IT-DMSES 6	0.134	0.018	−0.33	0.121
IT-DMSES 7	0.251	0.062	−0.74	0.003
IT-DMSES 8	0.267	0.071	−0.96	0.002
IT-DMSES 9	0.272	0.074	−0.83	0.002
IT-DMSES 10	0.071	0.005	−0.21	0.418
IT-DMSES 11	0.122	0.015	−0.39	0.159
IT-DMSES 12	0.151	0.023	−0.49	0.080
IT-DMSES 13	0.234	0.055	−0.67	0.007
IT-DMSES 14	0.16	0.026	−0.87	0.064
IT-DMSES 15	0.168	0.028	−0.86	0.051
IT-DMSES Total	0.159	0.025	−0.06	0.064
IT-DMSES Lifestyle Modification	0.282	0.080	−1.25	0.002
IT-DMSES Disease Management	0.271	0.074	−1.31	0.002

Note: IT-DMSES: Italian Version of the Diabetes Management Self-Efficacy Scale.

**Table 3:** Regression model for IT-DMSES 13 and BED adjusted for BMI, age, and gender.

	R	AIC	*p*
	0.502	550	<0.001
Independent Variables	β	SE	
BED 1–0	−3.1842	0.8833	<0.001
BMI	−0.0451	0.0298	0.133
Gender Female-Male	−1.0235	0.5133	0.049
Age	0.0552	0.0244	0.025

Note: BED: Binge Eating Scale; BMI: Body Mass Index.

## Data Availability

Raw data will be uploaded to www.zenodo.org immediately after the manuscript is accepted and will be available upon reasonable request from the authors, S.C. and A.G.U.

## Supplementary Materials

The following supporting information can be downloaded at: https://www.mdpi.com/article/10.3390/jcm15145626/s1, Table S1. Linear regression analysis for BMI and IT-DMSES items adjusted for age and gender.
