# Supplementary material for: Self-Efficacy Measured Through the Italian Diabetes Management Self-Efficacy Scale (IT-DMSES) in Individuals with Type 2 Diabetes
Source: J Clin Med. 2026 Jul 17;15(14):5626. doi: 10.3390/jcm15145626 (PMC13413273; doi:10.3390/jcm15145626)
Supplement: Supplementary file 1 [file jcm-15-05626-s001.zip › jcm-4374785-supplementary.pdf]

# Self-efficacy measured through the Italian Diabetes Management Self-Efficacy Scale (IT-DMSES) in individuals with type 2 diabetes

**Santo Colosimo[1,2*], Anna Guerrini Usubini[3], Sara Paola Mambrini[1,2], Gabriele Amodeo[4], Alessandro Sartorio[5], Gianluca Castelnuovo[3,6], Simona Bertoli[1,2].**

**Table S1.** Linear regression analysis for BMI and IT-DMSES items adjusted for age and gender.

| Item | R | $R^2$ | β | P value |
|---|---|---|---|---|
| **IT-DMSES 1** | 0.367 | 0.135 | -0.687 | 0.004 |
| **IT-DMSES 2** | 0.330 | 0.109 | -0.517 | 0.020 |
| **IT-DMSES 3** | 0.296 | 0.088 | -0.352 | 0.117 |
| **IT-DMSES 4** | 0.303 | 0.092 | -0.389 | 0.172 |
| **IT-DMSES 5** | 0.401 | 0.160 | -0.959 | 0.001 |
| **IT-DMSES 6** | 0.317 | 0.101 | -0.378 | 0.067 |
| **IT-DMSES 7** | 0.369 | 0.136 | -0.722 | 0.003 |
| **IT-DMSES 8** | 0.365 | 0.133 | -0.869 | 0.004 |
| **IT-DMSES 9** | 0.370 | 0.137 | -0.735 | 0.004 |
| **IT-DMSES 10** | 0.286 | 0.081 | -0.755 | 0.452 |
| **IT-DMSES 11** | 0.298 | 0.089 | -0.361 | 0.175 |
| **IT-DMSES 12** | 0.305 | 0.093 | -0.409 | 0.129 |
| **IT-DMSES 13** | 0.319 | 0.102 | -0.468 | 0.069 |
| **IT-DMSES 14** | 0.300 | 0.090 | -0.618 | 0.185 |
| **IT-DMSES 15** | 0.294 | 0.086 | -0.517 | 0.248 |
| **IT-DMSES Total** | 0.298 | 0.088 | -0.040 | 0.200 |
| **IT-DMSES Lifestyle Modification** | 0.372 | 0.138 | -1.080 | 0.006 |
| **IT-DMSES Disease Management** | 0.364 | 0.133 | -1.191 | 0.004 |
